# Experimental allergic encephalomyelitis in pituitary-grafted Lewis rats

**DOI:** 10.1186/1742-2094-3-20

**Published:** 2006-08-23

**Authors:** Ana I Esquifino, Pilar Cano, Agustín Zapata, Daniel P Cardinali

**Affiliations:** 1 Departamento de Bioquímica y Biología Molecular III, Facultad de Medicina, Universidad Complutense de Madrid, Spain; 2 Departamento de Biología Celular, Facultad de Ciencias Biológicas, Universidad Complutense de Madrid, Spain; 3 Departamento de Fisiología, Facultad de Medicina, Universidad de Buenos Aires, 1121 Buenos Aires, Argentina

## Abstract

Treatment of susceptible rats with dopaminergic agonists that reduce prolactin release decreases both severity and duration of clinical signs of experimental allergic encephalomyelitis (EAE). To assess to what extent the presence of an ectopic pituitary (that produces an increase in plasma prolactin levels mainly derived from the ectopic gland) affects EAE, 39 male Lewis rats were submitted to pituitary grafting from littermate donors. Another group of 38 rats was sham-operated by implanting a muscle fragment similar in size to the pituitary graft. All rats received subcutaneous (s.c.) injections of complete Freund's adjuvant (CFA) plus spinal cord homogenate (SCH) and were monitored daily for clinical signs of EAE. Animals were killed by decapitation on days 1, 4, 7, 11 or 15 after immunization and plasma was collected for prolactin RIA. In a second experiment, 48 rats were immunized by s.c. injection of a mixture of SCH and CFA, and then received daily s.c. injections of bromocriptine (1 mg/kg) or saline. Groups of 8 animals were killed on days 8, 11 or 15 after immunization and plasma prolactin was measured. Only sham-operated rats exhibited clinical signs of the disease when assessed on day 15 after immunization. A progressive decrease in plasma prolactin levels was observed in pituitary-grafted rats, attaining a minimum 15 days after immunization, whereas plasma prolactin levels were increased during the course of the disease in sham-operated rats. Plasma prolactin levels were higher in pituitary-grafted rats than in sham-operated rats 1 day after immunization, but lower on days 7, 11 and 15 after immunogen injection. Further supporting a correlation of suppressed prolactin levels with absence of clinical signs of EAE, rats that were administered the dopaminergic agonist bromocriptine showed very low plasma prolactin levels and did not exhibit any clinical sign of EAE. These results indicate that low circulating prolactin levels coincide with absence of clinical signs of EAE in Lewis rats.

## Findings

Experimental allergic encephalomyelitis (EAE) is one of best-studied models of autoimmune disease, and is characterized by an autoimmune attack on CNS myelin mediated by neural autoantigen-specific T helper cells [1]. EAE is currently the best available animal model of human multiple sclerosis. During induction of EAE, autoreactive T-cells are activated in the periphery by subcutaneous injection of either spinal cord homogenate (SCH) or CNS antigens, which may include myelin basic protein, myelin oligodendrocyte glycoprotein, or proteolipid protein or their peptides [2]. About 10 days after the combined injection of complete Freund adjuvant (CFA) and spinal cord homogenate (SCH), susceptible rats (e.g., Lewis rats) develop a progressive paralysis associated with CNS demyelinization. Activated, autoreactive T-cells access the CNS and, in the presence of competent antigen presenting cells, are further activated and induce a local inflammatory response. In most such models, the T helper 1 (Th1) subset of T-cells has been implicated in the induction phase of EAE.

It is well established that prolactin is an important modulator of the immune system through an effect that is exerted in part on the cellular arm of immune defense involving Th1 cytokines [3,4]. Factors that cause hypoprolactinemia are generally associated with reduced immunocompetence, whereas prolactin administration restores immunocompetence in hypophysectomized rats or bromocriptine-treated rats [5-7]. On the other hand, significant immune-suppressive effects of prolactin have been noticed: several investigators have reported that prolactin decreases natural killer cell migration and activity and reduces lymphocyte proliferative capacity and cytokine release in rodents [7-9]. Of relevance to EAE is the observation that treatment of animals with dopaminergic agonists which reduce prolactin release decreases both severity and duration of clinical signs of the disease [10,11].

Since changes in prolactin secretion could modulate EAE symptomatology, we wished to assess to what extent the presence of an ectopic pituitary, producing an increase in plasma prolactin levels mainly derived from the ectopic gland [12], would affect EAE. The consequence of inhibiting prolactin secretion by injecting the dopaminergic agonist bromocriptine was also assessed.

Male Lewis rats (6 weeks old, 140–170 g) were purchased from Charles River S.A., Spain, and were housed under standard conditions of controlled light (12:12 h light/dark schedule, light on at 0800 h) and temperature (22 ± 2°C). All experiments were conducted in accordance with the guidelines of the International Council for Laboratory Animal Science (ICLAS). Protocols were approved by the Institutional Animals Ethics Committee. Spinal cord, obtained from adult Wistar rats, was homogenized in PBS buffer at a concentration of 1 mg/ml to serve as an immunogen (SCH).

Two experiments were performed. In experiment 1, 39 rats were subjected to pituitary grafting from littermate donors. Animals were anesthetized with 2.5% tribromoethanol in saline (1 ml/100 g body weight). Another group of 38 rats of the same age was sham-operated (by implanting a muscle fragment of a size similar to the pituitary graft) to be used as a control group. Rats were left undisturbed for 4 weeks – i.e., until the time at which increased prolactin levels in pituitary grafted animals attains a plateau [13] – at the end of which time all animals were immunized by subcutaneous (s.c.) injection of a mixture of SCH and CFA containing Mycobacterium tuberculosis H37Ra (5 mg/ml; Difco Laboratories, Detroit, Michigan) (v/v) in a final volume of 200 μl. Animals were monitored daily for clinical signs of EAE using the following assessment scale: 0, normal; 0.5, loss of tonicity in distal half of tail; 1, piloerection; 2, total loss of tail tonicity; 3, hind leg paralysis; 4, paraplegia; and 5, moribund [14]. The rats were killed by decapitation on days 1, 4, 7, 11 or 15 after immunization (7–8 animals per group) and blood was collected from a trunk wound into heparinized tubes and was centrifuged. Plasma was collected and stored at -20°C until further analysis. Visual assessment of the grafts at the time of sacrifice did not show any gross differences between groups.

In experiment 2, 48 male Lewis rats were immunized with SCH plus CFA mixture, and then received daily s.c. injections of bromocriptine (1 mg/kg) or saline (n = 24 each). Animals were monitored daily for clinical signs of EAE and were killed by decapitation on day 8, 11 or 15 after immunization (8 animals per group), and blood was collected from a trunk wound.

Plasma prolactin levels were measured by a homologous specific double antibody RIA [15], using materials kindly supplied by the NIDDK's National Hormone and Pituitary Program and Dr. A. Parlow (Harbor UCLA Medical Center, Torrance CA 90509). The intra- and interassay coefficients were 6–8%. Sensitivity of the RIA was 40 pg/ml using the NIDDK rat prolactin RP-3 standard. Results were expressed as pg/ml of plasma. Statistical analysis of results was performed by employing either Student's t test, a factorial analysis of variance (ANOVA), or a one-way ANOVA followed by a Student-Newman-Keuls test.

**Figure 1:**
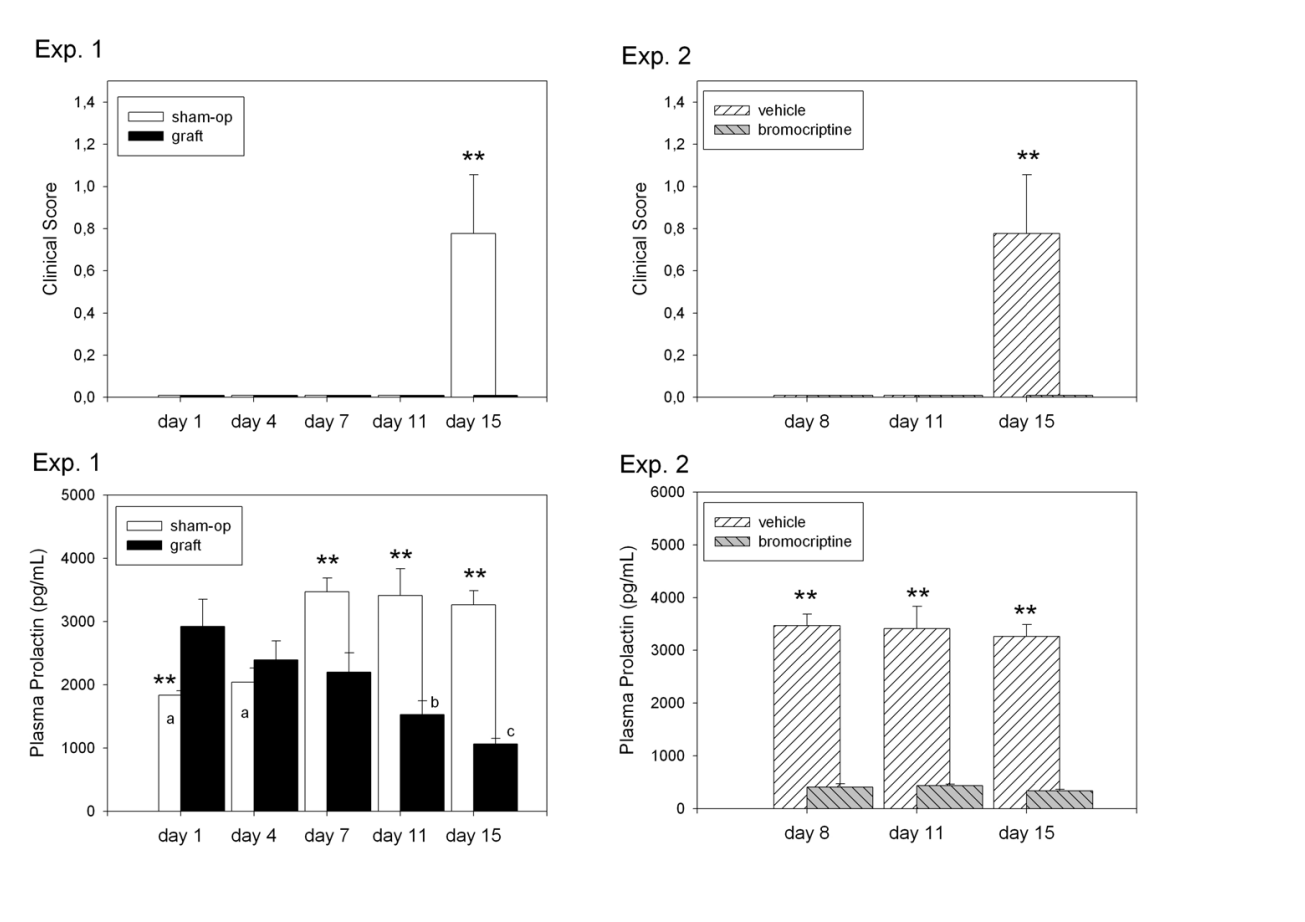
Effect of changing plasma prolactin levels on clinical signs of EAE in rats. Two experiments were performed. In experiment 1 (left upper and lower panels), 77 male Lewis rats were subjected either to pituitary grafting from littermate donors (n = 39) or to sham operations (n = 38); all were then immunized by s.c. injection of a mixture of spinal cord homogenate (SCH) and complete Freund's adjuvant (CFA) as described in Methods. Rats were monitored daily for clinical signs of EAE. Groups of 7–8 rats were killed by decapitation on day 1, 4, 7, 11 or 15 after immunization. In experiment 2 (right upper and lower panels), 48 male Lewis rats were immunized with SCH plus CFA mixture, and then received daily s.c. injections of bromocriptine (1 mg/kg) or saline (n = 24 each). Groups of 8 rats were killed by decapitation on day 8, 11 or 15 after immunization. Prolactin levels were measured by RIA. Data are shown as means ± SEM. ** p < 0.01 vs. grafted rats at each time interval, Student's t test. Superscripts designate significant differences among time intervals within the same experimental group, ^a ^p < 0.01 vs. sham-operated rats on days 7, 11 or 15; ^b ^p < 0.05 vs. pituitary-grafted rats on day 1; ^c ^p < 0.05 vs. pituitary-grafted rats on days 4 or 7, p < 0.01 vs. pituitary-grafted rats on day 1, one-way ANOVA, Student-Newman-Keuls test.

Figure 1 depicts the results of both experiments. The evolution of clinical scores of EAE in control and pituitary-grafted rats is shown in the upper left panel. Only sham-operated rats exhibited clinical signs of the disease when assessed on day 15 after immunization. Clinical scoring at this state of disease did not differ from that previously reported, using similar conditions, for both rats [16] and mice [17]. Prolactin levels in both experimental groups are shown in the lower left panel. A significant interaction "treatment × time" was found in the factorial ANOVA, i.e. plasma prolactin levels were higher in pituitary-grafted rats than in sham-operated rats 1 day after immunization and lower on days 7, 11 and 15 after immunogen injection (p < 0.001) (lower left panel). A progressive decrease in plasma prolactin levels was observed in pituitary-grafted rats, attaining a minimum 15 days after immunization (p < 0.01). In contrast, prolactin levels were increased during the course of the disease in sham-operated rats (p < 0.05). This increase in prolactin levels was presumably a response to the administration of CFA. Although the clinical onset of inflammatory disease following CFA occurs between days 12 and 15 after injection, the increase in plasma prolactin is already demonstrable at an early phase of the disease [18,19].

Mild hyperprolactinemia has been found to enhance several autoimmune diseases, including systemic lupus erythematosus, rheumatoid arthritis and autoimmune thyroiditis [6,7,20]. In studies of EAE, prolactin levels have been found to be elevated before the onset and during the disease [21], as observed in sham-operated animals in the present study. Therefore, a feasible explanation for the lack of clinical signs of EAE on day 15 after immunization in pituitary-grafted rats is a relative lack of prolactin.

Further supporting the correlation of suppressed prolactin levels with absence of clinical signs of EAE is the observation that rats administered the dopaminergic agonist bromocriptine did not exhibit any clinical sign of disease (Fig. 1, upper right panel). Bromocriptine treatment was very effective in preventing prolactin release at all examined time points (p < 0.001) (Fig. 1, lower right panel).

In summary, these results indicate that low circulating prolactin levels coincide with absence of clinical signs of EAE in Lewis rats. Since the presence of an ectopic pituitary would normally be expected to lead to increased plasma prolactin levels, further studies are needed to unravel why prolactin production by renal pituitary grafts is suppressed during EAE development. These results also confirm previous findings that treatment with dopaminergic agonists induces a reduction of prolactin levels accompanied by amelioration of the neurological signs of EAE [10,11]. The effects of dopaminergic agents could be related to their ability to lower prolactin concentrations and/or to a neuroprotective action of the drugs.

Caution should be taken in extrapolating these results to human disease as monophasic EAE, particularly in the Lewis rat model, shares only some aspects of human multiple sclerosis. Indeed, contradictory results have been published on the occurrence of hyperprolactinemia in multiple sclerosis patients, with some studies finding increased prolactin levels [22-24] while others do not [25-27].

## Competing interests

The author(s) declare that they have no competing interests.

## Authors' contributions

AIE, PC and AZ carried out the experiments and the immunoassays. DPC and AIE designed the experiments. DPC also performed the statistical analysis and drafted the manuscript. All authors read and approved the final manuscript.
